# Accuracy and reliability of the Pfeffer Questionnaire for the Brazilian elderly population

**DOI:** 10.1590/1980-57642015DN92000012

**Published:** 2015

**Authors:** Marina Carneiro Dutra, Raynan dos Santos Ribeiro, Sarah Brandão Pinheiro, Gislane Ferreira de Melo, Gustavo de Azevedo Carvalho

**Affiliations:** 1 Post-graduate Program in Gerontology, Catholic University of Brasília (UCB), Brasília Campus DF, Brazil.; 2 Physiotherapy Course; Catholic University of Brasília (UCB), Brasília campus DF, Brazil.; 3 Post-graduate Program in Gerontology; UCB, Brasília DF, Brazil.; 4 PhD in Physical Education, Catholic University of Brasília (UCB); Professor on Post-graduate Stricto Sensu Program in Gerontology (UCB), Brasília Campus DF, Brazil.; 5 PhD in Health Sciences, Catholic University of Brasília (UCB); Professor on Post-graduate Stricto Sensu Program in Gerontology (UCB), Brasília Campus DF, Brazil.

**Keywords:** accuracy, reliability, functional ability, cognition, elderly

## Abstract

The aging population calls for instruments to assess functional and cognitive impairment in the elderly, aiming to prevent conditions that affect functional abilities.

**Objective:**

To verify the accuracy and reliability of the Pfeffer (FAQ) scale for the Brazilian elderly population and to evaluate the reliability and reproducibility of the translated version of the Pfeffer Questionnaire.

**Methods:**

The Brazilian version of the FAQ was applied to 110 elderly divided into two groups. Both groups were assessed by two blinded investigators at baseline and again after 15 days. In order to verify the accuracy and reliability of the instrument, sensitivity and specificity measurements for the presence or absence of functional and cognitive decline were calculated for various cut-off points and the ROC curve. Intra and inter-examiner reliability were assessed using the Interclass Correlation Coefficient (ICC) and Bland-Altman plots.

**Results:**

For the occurrence of cognitive decline, the ROC curve yielded an area under the curve of 0.909 (95%CI of 0.845 to 0.972), sensitivity of 75.68% (95%CI of 93.52% to 100%) and specificity of 97.26%. For the occurrence of functional decline, the ROC curve yielded an area under the curve of 0.851 (95%CI of 64.52% to 87.33%) and specificity of 80.36% (95%CI of 69.95% to 90.76%). The ICC was excellent, with all values exceeding 0.75. On the Bland-Altman plot, intra-examiner agreement was good, with p>0.05consistently close to 0. A systematic difference was found for inter-examiner agreement.

**Conclusion:**

The Pfeffer Questionnaire is applicable in the Brazilian elderly population and showed reliability and reproducibility compared to the original test.

## Abstract (pt)

Com o envelhecimento populacional, ocorre a necessidade de instrumentos adequados para avaliar o comprometimento funcional e cognitivo do idoso, visando prevenir qualquer condição que afete sua funcionalidade.

**Objetivo:**

Verificar a acurácia e confiabilidade da escala de Pfeffer (FAQ) para a população idosa brasileira; avaliar a confiabilidade e reprodutibilidade da versão traduzida do questionário de Pfeffer.

**Métodos:**

A versão brasileira do FAQ foi aplicada em 110 idosos divididos em dois grupos. Ambos passaram, em um primeiro momento e após 15 dias, por uma avaliação feita por dois pesquisadores, de forma cega. Para verificar a acurácia e confiabilidade do instrumento, medidas de sensibilidade e especificidade para a ocorrência ou não de declínio funcional e cognitivo foram calculadas para diversos pontos de corte e a curva ROC. Para medir a confiabilidade intra e interexaminadores foram utilizados o Coeficiente de Correlação Interclasse (ICC) e o gráfico Bland-Altman.

**Resultados:**

Para a ocorrência de declínio cognitivo a curva ROC forneceu uma área sob a curva igual a 0,90, sensibilidade igual a 75,68% e especificidade de 97,26%. Para a ocorrência de declínio funcional, a curva ROC forneceu uma área sob a curva igual a 0,851, sensibilidade igual a 75,93% e especificidade de 80,36%. O ICC foi excelente, sendo todos os valores superiores a 0,75. No gráfico Bland-Altman, a concordância intraexaminadores foi boa, já na concordância interexaminadores houve uma diferença sistemática.

**Conclusão:**

O questionário de Pfeffer tem aplicabilidade na população idosa brasileira e apresenta confiabilidade e reprodutibilidade quando comparado ao teste original.

## INTRODUCTION

Improvements in healthcare and socioeconomic conditions have contributed to an increase in longevity of the population, which is associated with a greater prevalence of chronic diseases, functional dependence and decline in cognitive abilities.^1^

Although changes in cognitive performance occur in some domains with aging, these impairments often do not affect the daily lives of elderly and their family members. However, when the decline is greater than expected for the individual's age and schooling, this is defined as Mild Cognitive Impairment (MCI). MCI is characterized by memory complaints and memory impairment on tests, yet with preserved global cognitive function and no dementia.^2-4^

Assessment of cognitive functions can allow early detection of individuals in this situation, allowing the elder and their family to take steps toward averting or delaying the manifestation of the social and emotional upheaval which the development of a dementia can cause.^5^

Cognitive decline can affect the occupational functioning of elderly, i.e. the ability to carry out everyday activities. These activities include the so-called instrumental activities of daily living (IADL), whose independence for performance is directly linked to the ability for independent living in the community.^6,7^

Akin to cognitive assessment, assessing the functional status of the elderly is of utmost importance to allow the adoption of the most adequate treatment or preventive conduct. Such assessments must be performed using adequate, accurate instruments. One such instrument widely used in the clinical assessment and longitudinal follow-up of elderly is the Functional Assessment Questionnaire – FAQ, developed by Pfeffer et al.,^8^ in 1982.

The Pfeffer questionnaire or FAQ, is widely used in international studies and constitutes a 10-item instrument for determining functioning based on the level of independence in performing IADLs. Each item is scored on a scale of 0 (independence) to 3 (dependence), where higher scores reflect greater dependency of the patient. Applied alone, the FAQ is useful for assessing IADLs, and when used in combination with the Mini-Mental State Exam – MMSE, it can assess cognitive decline with greater specificity.^9^

In 2011, the Pfeffer questionnaire was translated for use in the Brazilian population by Sanchez et al.^10^ The instrument underwent translation and back-translation, and the test-retest reliability of a version proposed for use in Brazil was analyzed; the results of the study suggested that the adapted version of the questionnaire is a reliable instrument applicable for assessing the functioning of Brazilian older adults.

The aim of the present study was to assess the accuracy and reliability of the translated version for use in the elderly population. In order to ascertain the accuracy and ability of the method for reaching a correct diagnosis, the specificity and sensitivity of the test was investigated. Reliability in the protocol was also determined, which was supported and ratified by the study of Sanchez et al. (2011), by identifying intra and interexaminer reproducibility for the model and population studied.

Sensitivity indicates the ability of a test to correctly detect individuals with a disease/condition, while specificity indicates the ability of a test to correctly exclude those without the disease/condition, both of which were assessed in the present study using the ROC (Receiver Operating Characteristic) curve.

In summary, although numerous instruments for assessing the functional performance of elderly are available, few have been adapted for use in the Brazilian population.^11^ Therefore, the triad of cognitive impairment, functional performance, and assessment, is of fundamental importance for monitoring elderly, calling for adequate instruments to assess this specific group.

## METHODS

A cross-sectional study was conducted to determine the accuracy and reliability of an assessment instrument developed at the Unidade Mista de Taguatinga (UMT/DF), a public institution with a referral center for elderly care. For the study, elderly diagnosed with mild cognitive impairment were selected from the center whereas community-dwelling elderly without cognitive impairment were recruited from their homes.

The sample population included 110 elderly, based on a sample-size calculation, divided into two groups: Group one comprising 73 elderly without cognitive decline (G1); and Group two comprising 37 older adults with cognitive decline (G2). Data collection was carried out between October 2013 and January 2014, and the assessment protocol took an average of 15 minutes to apply to the caregiver and 30 minutes to the elderly individual.

Elderly patients with clinically-confirmed diagnosis of mild cognitive decline were selected from the UMT/DF by reviewing medical records. This center recognizes the criteria of Petersen^12^ for diagnosing mild cognitive impairment (MCI).

The inclusion criteria for G1, comprising elderly exhibiting no cognitive decline on the MMSE, were as follows: being aged 60 years or older; attaining MMSE scores compatible with schooling level; thus not exhibiting cognitive decline according to the specific criteria^13^ of 20 points for illiterates, 25 points for elderly with 1-4 years of schooling, 26.5 points for elderly with 5-8 years, 28 points for 9-11 years, and 29 points for those with 11 or more years of schooling. The inclusion criteria for G2, comprising elderly diagnosed with MCI, were as follows: being aged 60 years or older; having a clinical diagnosis of mild cognitive impairment.

The exclusion criteria for both groups were: having a diagnosis of depression, dementias, neurological or orthopedic diseases, use of walking aids (for instance, walker or cane); and elderly who, in the 15-day period since the baseline assessment, had any medical complication that changed their initial health status.

The project was first submitted to the Research Ethics Committee of the Catholic University of Brasília – UCB, and also to the Foundation for Teaching and Research in Health Sciences (FEPECS) and was approved under process number 19391513.7.0000.0029. Data collection commenced following approval of the study was granted.

After obtaining authorization by the institution where the study was conducted, the sample was screened according to the inclusion and exclusion criteria proposed. The study objectives were explained at the time of requesting participation in the study. Elderly agreeing to take part in the study were asked to sign the Free and Informed Consent Form. The elderly were informed about the possibility of being dropped from the study, in the event that any of the exclusion criteria were met during data collection, and also that they could withdraw from the study at any time.

The team of researchers consisted of the lead researcher who was a physiotherapist, and an assistant, also a physiotherapist. Prior to data collection, the researchers studied the subject together and trained on applying the tests in order to harmonize the collection procedure. For this purpose, a pilot study involving 13 elderly from G1 was performed, with these participants subsequently included in the sample.

The instruments were applied to the elderly in the following order:

[1] Sociodemographic Questionnaire to characterize the study sample;
[2] MMSE for cognitive screening;
[3] Lawton and Brody Scale for functional assessment;
[4] Translated version of the Pfeffer Questionnaire applied to an informant (family member, companion or caregiver).

The application of the tests, along with their objectives, was explained individually to each participant. Initial collection (Sociodemographic Questionnaire, MMSE and Lawton & Brody) was carried out by only one of the examiners, whereas the FAQ was applied by both examiners at the first (baseline) and second assessments.

In the present study, the accuracy and reliability of the translated instrument was determined by comparing against data derived from widely recognized and accepted cognitive and functional assessment parameters, in this case the MMSE and the Lawton & Brody scale, respectively.

In order to evaluate intra and inter-examiner reliability, the 110 elderly were assessed by the researchers initially at the first timepoint. Subsequently, at the second timepoint after a 15-day period, the participants were assessed again by the two researchers independently, so as to avoid response bias or changes in the initial status of the elderly. The second assessment was performed by telephone, where two calls were made to the interviewee on the same day at different times. The examiners were blinded to the results collected at the two assessments. The same order of examiners was maintained for application of the tests.

The intraclass correlation coefficient (ICC) was calculated, with the lower limit of the 95% interval, obtained by analysis of variance with a one classification criterion. Values of ICC exceeding 0.75% were deemed to indicate excellent agreement.^14^ The Bland-Altman method was also used, which entails plotting a graph of the difference between measurements against mean measurements. The method assesses the degree of disagreement (including systematic differences), discrepant points, and the occurrence of tendency.

Sensitivity and specificity measurements for the occurrence or otherwise of functional and cognitive decline, respectively, were calculated for several cut-off points and the ROC curve was subsequently built. The values were considered optimal the closer they were to 1. Sensitivity and specificity measurements for cognitive decline were also calculated, along with the respective 95% confidence intervals for the optimum cut-off point.

## RESULTS

The sample comprised 110 elderly residing in the Federal District, 76.4% (n=84) women and 23.6% (n=26) men, with mean age of 71.51 years.

In group 1, only 2 participants were excluded from the study, for attaining less-than-expected scores on the MMSE for their level of schooling.

In group 2, a total of 91 medical records of patients diagnosed with MCI were reviewed, 62 of which met the inclusion criteria established for the study. After this review, the patients were contacted by telephone and invited to take part in the study. Eleven patients could not be located, 5 refused to participate in the study and 9 did not show up for the assessment, even after a second contact, thereby giving a final study sample of 37 elderly.

**Figure 1:**
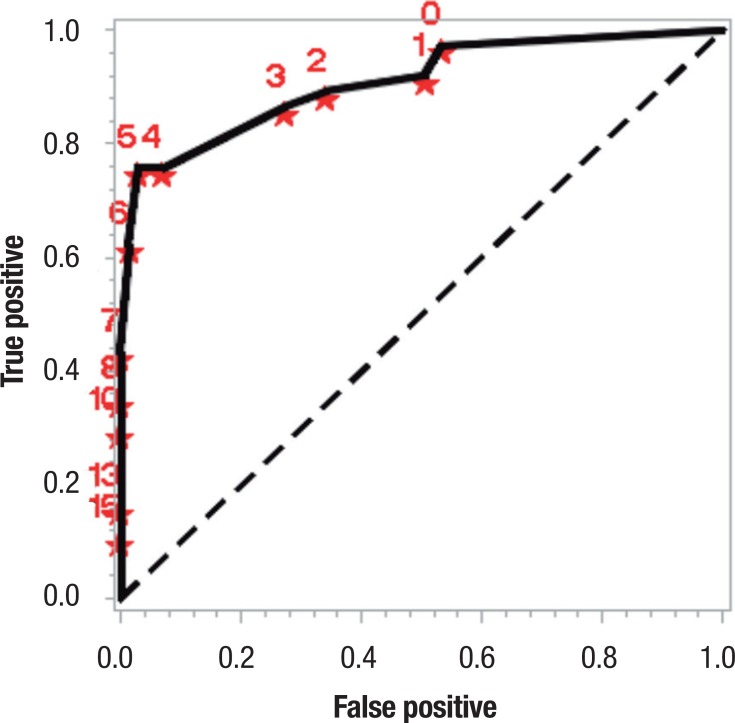
ROC curve for occurrence of cognitive decline.

Based on several cut-off points, a ROC curve was produced for the occurrence of cognitive decline, yielding an area under the curve of 0.909 (95%CI of 0.845 to 0.972), as shown in Figure 1. The sensitivity tested was 75.68% with 95%CI limits of 61.85% and 89.50% while specificity was 97.26% with 95%CI limits of 93.52 to 100%.

**Figure 2:**
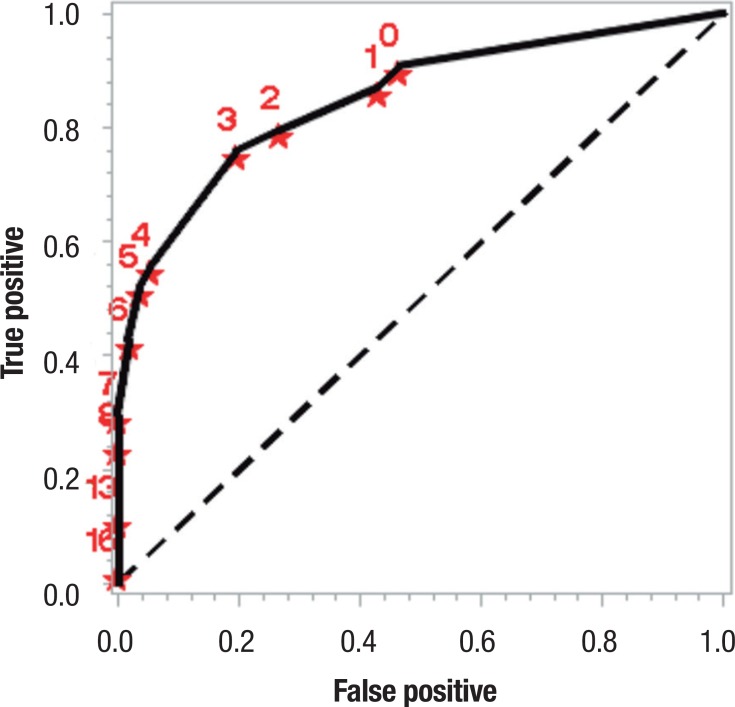
ROC curve for occurrence of functional decline.

Similarly, based on several cut-off points, another ROC curve was produced for the occurrence of functional decline, obtained through the correlation of the FAQ with the Lawton & Brody scale, yielding an area under the curve of 0.851 (95%CI of 0.778 to 0.923), as shown in Figure 2. The sensitivity tested was 75.93% with 95%CI limits of 64.52% and 87.33% while specificity was 80.36% with 95%CI limits of 69.95 and 90.76%.

The ICC for inter-examiners and intra-examiners was excellent, with the highest scores obtained on the inter-examiner assessment (1 and 0.999). All lower limits of the 95% confidence interval were well above the value of 0.75.

The intra-examiner measurements exhibited lower reproducibility than interexaminer measurements, as shown in Table 1.

**Table 1:** Intra and Inter-examiner Correlation Coefficient for the FAQ.

Intraexaminer agreement	ICC	
	Examiner 1	Examiner 2
All patients	0.967 (0.952)	0.958 (0.939)
G1	0.903 (0.849)	0.908 (0.858)
G2	0.951 (0.906)	0.928 (0.865)
**Interexaminer agreemnt**	**1^st^ Assessment**	**2^nd^ Assessment**
All patients	0.995 (0.992)	0.999 (0.999)
G1	0.970 (0.952)	1
G2	0.977 (0.994)	0.997 (0.995)

ICC: Intra/Interexaminer correlation coefficient with 95% confidence lower limit (parentheses).

**Figure 3:**
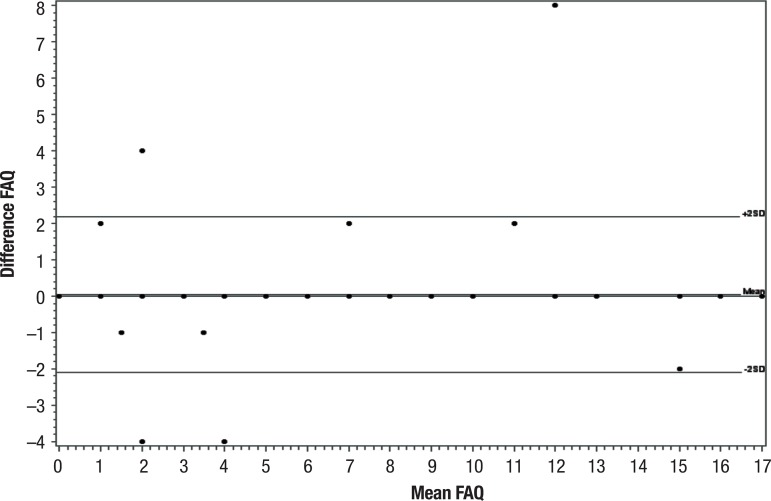
Bland-Altman 1 – Intra-examiner agreement (examiner 1 in all patients).

**Figure 4:**
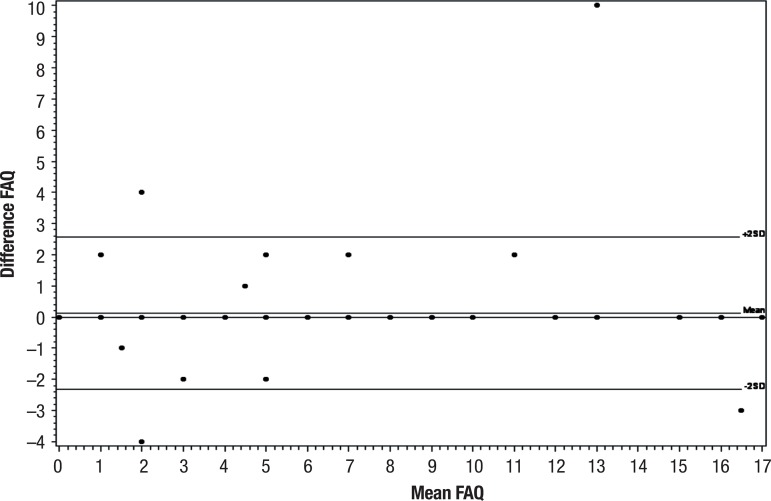
Bland-Altman 1 – Intra-examiner agreement (examiner 2 in all patients).

These results are congruent with the Bland-Altman plots (Figures 3 and 4), where no statistically significant bias can be observed, i.e. that there was no statistically significant difference between the assessments. For interexaminer agreement, the graph for the second assessment was not produced given agreement was 100%.

## DISCUSSION

The Pfeffer questionnaire, or FAQ, underwent translation and back-translation, and the test-retest reliability of a version proposed for use in Brazil was analyzed. The results of the study suggested that the adapted version of the questionnaire is a reliable and stable instrument and is applicable for assessing the functioning of Brazilian older adults.^10,11^

The accuracy and reliability of an instrument helps professionals determine the objectives of treatment, as well as to assess its effects and predict risks.^16^ This information is has also been verified and exploited by other studies.^17,18^

In the study conducted by Sanchez et.al., after carrying out the first pre-test in a target population, the instrument was adjusted to correspond with the interviewee's perception and with the assumed meaning of the items.^19^ In the present study, the version used allowed a standardized and objective assessment of functioning.

The FAQ is an instrument suited for assessing loss of functional ability of individuals.^20-22^ No conflicting information was found the literature regarding the test's reliability or ease-of-application.

In order to verify the accuracy and reliability of an instrument, comparisons must be drawn against results derived from another recognized and accepted instrument for the assessment in question. In the present study, the results obtained on the FAQ were compared against those of the MMSE and the Lawton & Brody scale.

When compared to the MMSE, the cut-off point found in the present study corroborated the cut-off point of the original study performed by Pfeffer et al. (1982). Another factor mirrored by the two studies was that the instrument had greater specificity (97.26%) than sensitivity (75.68%).

The area under the curve yielded a value of 0.909, indicating that the FAQ is a good instrument for screening cognitive decline and, although no studies were found to compare the results, the literature reports that the instrument is widely used in international studies. In Brazil, the questionnaire is recommended by the Brazilian Academy of Neurology as an instrument for the diagnosis of functional decline in cases of suspected dementia, and is used in international studies conducted by the Pan-American Health Organization.^9,22^

The original study suggested that the FAQ, applied in conjunction with a cognitive assessment test, could distinguish normal from demented elderly, making it potentially useful for diagnosing cognitive decline. This information has also been exploited by other authors.^11,20,21^ In this regard, results of studies on instruments for diagnosing dementia have shown that the MMSE combined with functional assessment instruments, such as the FAQ, substantially improved diagnostic accuracy compared to the performance of the instruments when used alone.^22^

Compared to the Lawton & Brody scale, the cut-off point obtained in the present study differed to that reported in the literature. The optimum cut-off point found was the occurrence of functional decline for scores > 3. This finding may be explained by the fact that the 17 elderly from G1, whose MMSE performance indicated no cognitive decline and had scores < 6 on the FAQ assessment, were classified as dependent on the Lawton & Brody scale. The elderly obtained a score of 20 and all answered stating they required assistance on the item "only for performing heavy domestic tasks".

In the original study conducted by Pfeffer, the FAQ proved more sensitive (0.85) than the Lawton & Brody scale (0.57), and almost as specific (0.81 and 0.92, respectively), for distinguishing normal individuals and those with cognitive decline.

Assessment instruments should be reproducible, in other words they must replicate equal or similar results following two or more administrations to the same patient, provided their initial health status has not changed.^10,23-25^ The intra and interexaminer reproducibility and reliability in this study was excellent, as measured by the ICC. In analyses involving all patients, as well as on a group level, the ICC was well above 0.75, corroborating the results found in the studies of Sanchez et al.; interviews were repeated after 15-60 days, with a mean interval of 32 days between the applications (SD=12.85) and the ICC was 0.97.

The Bland-Altman method produced a figure on which the size and amplitude of the difference in means and errors or outliers can be readily interpreted. The plot also shows confidence interval values for the difference in mean and agreement limits, necessary information on which to ground clinical decisions.^26^

Thus, good agreement was observed for the FAQ analysis on Bland-Altman plots, with no statistically significant bias, i.e. no statistically significant difference between the assessments and no departures from zero on the horizontal line across all assessments, where p>0.05remained close to zero. This showed that the assessments had good agreement, with the exception of a few points outside the limits of agreement.

However, on the first assessments conducted by examiner 1 and examiner 2 in group 1, a systematic difference was observed, i.e. a small difference (0.1) between the assessments, evidenced by departure from the zero of the horizontal line. Thus, the value of the test measured by examiner 1 tended to be highly similar to that measured by examiner 2. With a value of p=0.0324, the spatial distribution of the points is homogenous, not indicating a relationship between the differences and the mean measurements.

The fact that the FAQ has not yet been translated and adapted for other countries and languages, precluded comparison with results in other cultures.

With regard to operational equivalence, good agreement was observed in the second assessment conducted by telephone, corroborating the results of Sanchez et al.,^11^ where administration by telephone was tested and the reliability (0.95) suggested the test version had not led to changes in item consistency, showing good reproducibility.

In conclusion, the Brazilian version of the Pfeffer (FAQ) questionnaire exhibited strong correlation with the MMSE and the Lawton & Brody scale, instruments assessing cognitive state and functional ability, respectively. Analysis of the ROC curve showed that the FAQ is a good instrument for functional assessment and for screening cognitive decline.

The instrument had greater specificity than sensitivity for both cognitive and functional assessment. The reproducibility of the translated version was excellent and the Interclass Correlation Coefficient (ICC) values calculated were well over 0.75, with the highest values found for interexaminer assessment. The Bland-Altman plots also showed good agreement on intra and interexaminer assessments.

The FAQ is applicable in the Brazilian elderly population and has good accuracy and reliability. Its ease of application makes the instrument practical for use in research settings and clinical practice.
